# Correction: Family nurture intervention (FNI): methods and treatment protocol of a randomized controlled trial in the NICU

**DOI:** 10.1186/1471-2431-12-107

**Published:** 2012-07-25

**Authors:** Martha G Welch, Myron A Hofer, Susan A Brunelli, Raymond I Stark, Howard F Andrews, Judy Austin, Michael M Myers, FNI Trial Group

**Affiliations:** 1 Department of Psychiatry, College of Physicians & Surgeons, 1051 Riverside Drive, Unit 40, New York, NY, 10032, USA; 2 Department of Pediatrics, College of Physicians & Surgeons, 630 West 168th Street, New York, NY, 10032, USA; 3 Department of Pathology & Cell Biology, College of Physicians & Surgeons, 630 West 168th Street, New York, NY, 10032, USA; 4 Mailman School of Public Health, Columbia University, 722 West 168th Street, New York, NY, 10032, USA; 5 Department of Psychiatry, New York State Psychiatric Institute, 1051 Riverside Drive, New York, NY, 10032, USA; 6 Department of Psychology, Bronfman Science Center, Williams College, 18 Hoxsey Street, Williamstown, MA, 01267, USA

## 

Authors Correction:

^1, §^ Department of Psychiatry, College of Physicians & Surgeons, 1051 Riverside Drive, Unit 40, New York, NY, 10032, USA

Text correction:

In our methods paper [1], we misstated the primary outcome variable, In addition, the power analyses on which the study Ns are based was not included in the article.

(1) The single primary outcome variable of our study design was Length of Stay (LOS), the number of days in the intensive care unit from day of birth to day of discharge home. All measures described in the paper as primary outcomes are secondary outcomes, including those outcomes listed in Tables 1 and 2.

(2) Previous studies that used LOS for evaluating interventions had total sample sizes of N = 128, 100, and 28, respectively [2-4]. To be conservative, we designated a total N = 150 to increase the prospect of statistical significance within a more acute population from our NICU (lower average birth weight and age) and to allow for attrition (including transfer to local level 2 NICUs). It was calculated that, for a significance level of alpha = .05, one hundred fifty infants would be sufficient to provide 80% power to detect a 5.3 day difference in length of hospital stay.

## Pre-publication history

The pre-publication history for this paper can be accessed here:

http://www.biomedcentral.com/1471-2431/12/107/prepub
