# Supplementary figures and images for: Family nurture intervention (FNI): methods and treatment protocol of a randomized controlled trial in the NICU
Source: BMC Pediatr. 2012 Feb 7;12:14. doi: 10.1186/1471-2431-12-14 (PMC3394087; doi:10.1186/1471-2431-12-14)

PATIENT # \_\_\_\_\_

DATE \_\_\_\_\_

# MOM'S SUPPORT CIRCLE

WHO WILL SUPPORT YOU WITH YOUR BABY WHEN YOU GET HOME?

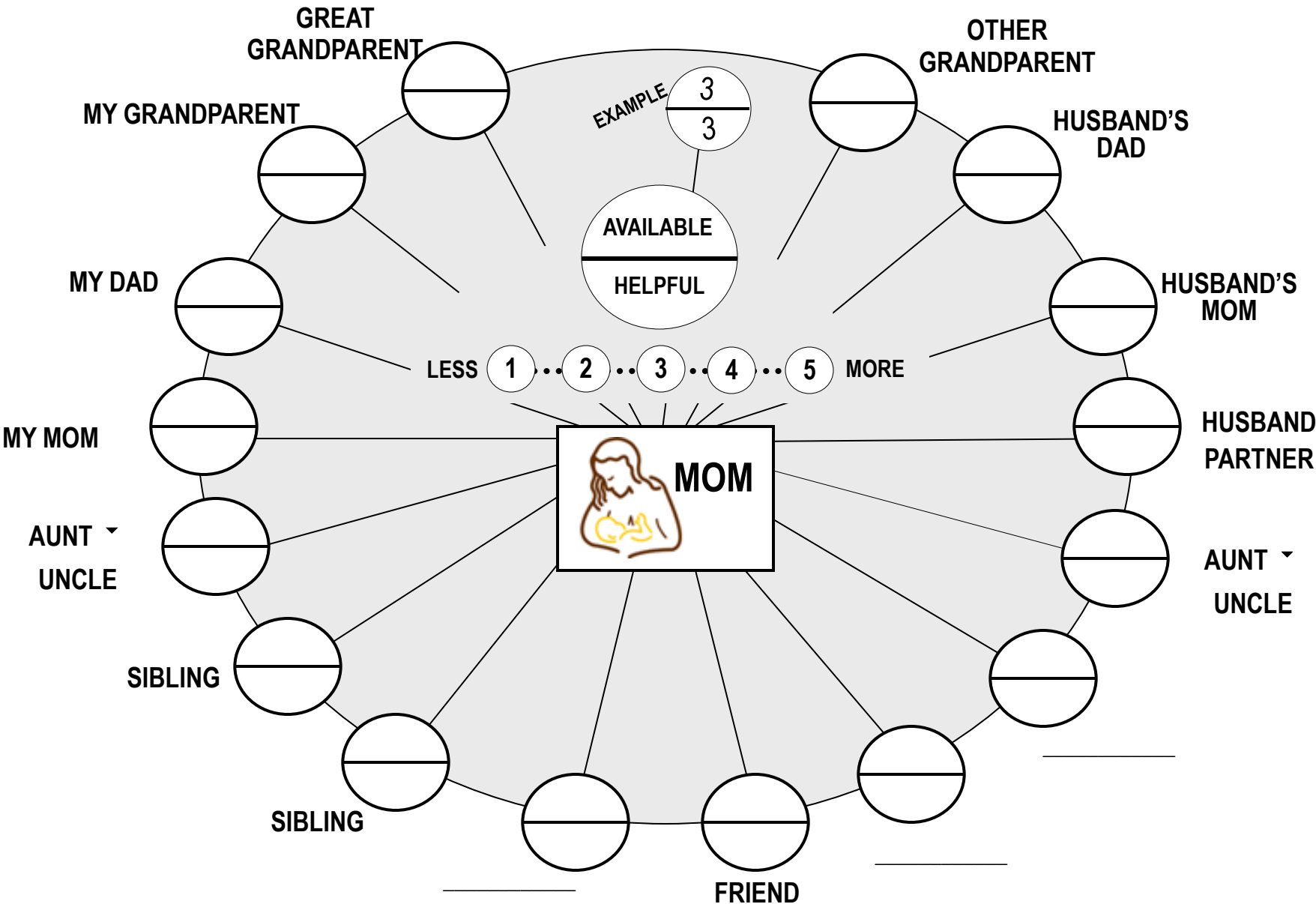

Supplement: Additional file 1 — Mom's Support Circle. [file 1471-2431-12-14-S1.PDF]
